# Prediction of TKI response in EGFR-mutant lung cancer patients-derived organoids using malignant pleural effusion

**DOI:** 10.1038/s41698-024-00609-7

**Published:** 2024-05-21

**Authors:** Sang-Hyun Lee, Kyuhwan Kim, Eunyoung Lee, Kyungmin Lee, Kyeong Hwan Ahn, Hansom Park, Yelim Kim, Soeun Shin, Sang Youl Jeon, Yongki Hwang, Dong Hyuck Ahn, Yong-Jun Kwon, Seok Whan Moon, Mi Hyoung Moon, Kyung Soo Kim, Kwanyong Hyun, Tae-Jung Kim, Yeoun Eun Sung, Joon Young Choi, Chan Kwon Park, Sung Won Kim, Chang Dong Yeo, Hyun-Jung Sohn, You-Seok Hyun, Tai-Gyu Kim, Bosung Ku, Jeong Uk Lim, Seung Joon Kim

**Affiliations:** 1 Precision Medicine Research Institute, Medical & Bio Decision (MBD) Co., Ltd., Suwon, Republic of Korea; 2 Division of Pulmonology, Department of Internal Medicine, Seoul St. Mary’s Hospital, College of Medicine, The Catholic University of Korea, Seoul, Republic of Korea; 3 Translational Medicine Operations Hub, Luxembourg Institute of Health, Dudelange, Luxembourg; 4 Department of Thoracic and Cardiovascular Surgery, College of Medicine, The Catholic University of Korea, Seoul, 06591 Republic of Korea; 5 Department of Hospital Pathology, College of Medicine, The Catholic University of Korea, Seoul, 06591 Republic of Korea; 6 Division of Pulmonary and Critical Care Medicine, Department of Internal Medicine, Incheon St. Mary’s Hospital, College of Medicine, The Catholic University of Korea, Seoul, Republic of Korea; 7 Division of Pulmonary, Critical Care and Allergy, Department of Internal Medicine, Yeouido St. Mary’s Hospital, College of Medicine, The Catholic University of Korea, Seoul, Republic of Korea; 8 Department of Otorhinolaryngology-Head and Neck Surgery, College of Medicine, The Catholic University of Korea, Seoul, Republic of Korea; 9 Department of Biomedicine & Health Sciences, College of Medicine, The Catholic University of Korea, Seoul, Republic of Korea; 10 Division of Pulmonary and Critical Care Medicine, Department of Internal Medicine, Eunpyeong St. Mary’s Hospital, College of Medicine, The Catholic University of Korea, Seoul, Republic of Korea; 11 ViGenCell Inc., Seoul, Republic of Korea; 12 Postech-Catholic Biomedical Engineering Institute, Songeui Multiplex Hall, College of Medicine, The Catholic University of Korea, Seoul, Republic of Korea

**Keywords:** Oncology, Lung cancer

## Abstract

Patient-derived organoids (PDOs) are valuable in predicting response to cancer therapy. PDOs are ideal models for precision oncologists. However, their practical application in guiding timely clinical decisions remains challenging. This study focused on patients with advanced EGFR-mutated non-small cell lung cancer and employed a cancer organoid-based diagnosis reactivity prediction (CODRP)-based precision oncology platform to assess the efficacy of EGFR inhibitor treatments. CODRP was employed to evaluate EGFR-tyrosine kinase inhibitors (TKI) drug sensitivity. The results were compared to those obtained using area under the curve index. This study validated this index by testing lung cancer-derived organoids in 14 patients with lung cancer. The CODRP index-based drug sensitivity test reliably classified patient responses to EGFR-TKI treatment within a clinically suitable 10-day timeline, which aligned with clinical drug treatment responses. This approach is promising for predicting and analyzing the efficacy of anticancer, ultimately contributing to the development of a precision medicine platform.

## Introduction

The effectiveness of precision oncology depends on models that encompass the morphological, molecular, and functional traits of tumors, enabling precise prediction of drug response and resistance. The development of diverse patient-derived cancer models (PCMs) are invaluable resources in this regard. Researchers have used PCM in drug sensitivity assays to successfully replicate the antitumor responses observed in clinical settings, highlighting its reliability in guiding personalized treatment approaches^1,2^.

Despite the potential of PCMs such as patient-derived cell lines and patient-derived xenografts (PDXs), there are considerable technical limitations that hinder their clinical translation for drug sensitivity assays and decision-making. Patient-derived cell lines can undergo genetic and morphological changes over time, rendering them unsuitable for reliable clinical screening^3,4^. Although PDXs retain intratumoral clonal architecture and genetic diversity, establishing them is expensive and time-consuming, limiting their applicability in diagnostic drug screening and precision medicine^5,6^.

In comparison, patient-derived organoids (PDOs) offer cost-effective high-throughput models for clinical applications. With the availability of large-scale biobanks for various cancer types, PDOs have demonstrated the ability to capture patient diversity. They have been successfully used in broad-based drug screening to reproduce known associations between genetic mutations and sensitivity to targeted therapies. As a result, PDOs hold promise as a functional precision medicine technology for guiding treatment decisions^2,7^. However, challenges exist in expanding the number of PDOs for drug screening within the timeframe typically required for treatment decisions (within 14 days of diagnosis). The current PDO generation processes are slow and inefficient, resulting in unacceptable treatment delays. To develop a clinically useful diagnostic assay, it is essential to accelerate PDO generation and functional testing as well as to develop automated procedures from a core biopsy.

To overcome the aforementioned technical challenges, we have developed the cancer organoid-based diagnosis reactivity prediction (CODRP) platform, which includes a cell culture chip (Cellvitro® 384 pillar/well plate, MBD Co., Ltd.)^8^ capable of culturing cells at the nanoliter level, a cell dispenser (ASFA® spotter, MBD Co., Ltd.)^8,9^ within a hydrogel for precise and rapid cell seeding, a fluorescence scanner (ASFA® scanner, MBD Co., Ltd.)^10^ for measuring drug responses, and algorithms (CODRP PhenoSW, MBD Co., Ltd.) to enhance correspondence with clinical outcomes. In this study, we established a methodology for obtaining a large number of LCOs from pleural effusion (PE) samples from patients with lung cancer. We confirmed that LCOs maintained their morphological and histological characteristics consistent with those of the original tumors. The nanoliter scale of the pillar chips allowed the testing of an array of clinically recommended drugs with P0-derived LCOs alone, eliminating the need for prolonged expansion and enabling drug response evaluation within 1 week. Our evaluation of drug responses in LCOs using the CODRP index demonstrated a significant improvement in matching clinical outcomes compared to the conventional area under the curve (AUC) values used for drug response assessment. We anticipate that the CODRP platform will offer an efficient method for prompt prediction of patient-specific drug responses in lung cancer.

## Results

### Cell isolation from pleural effusion

**Fig. 1:**
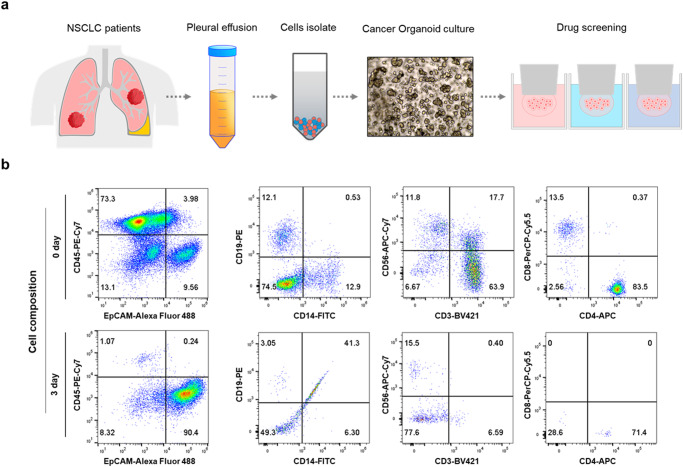
In the PE of lung cancer patients, various types of cells are present. **a** A schematic representation of cell separation from pleural effusion, followed by organoid culture and drug screening process. **b** Identification of cell types present in the PE through FACS analysis.

**Fig. 2:**
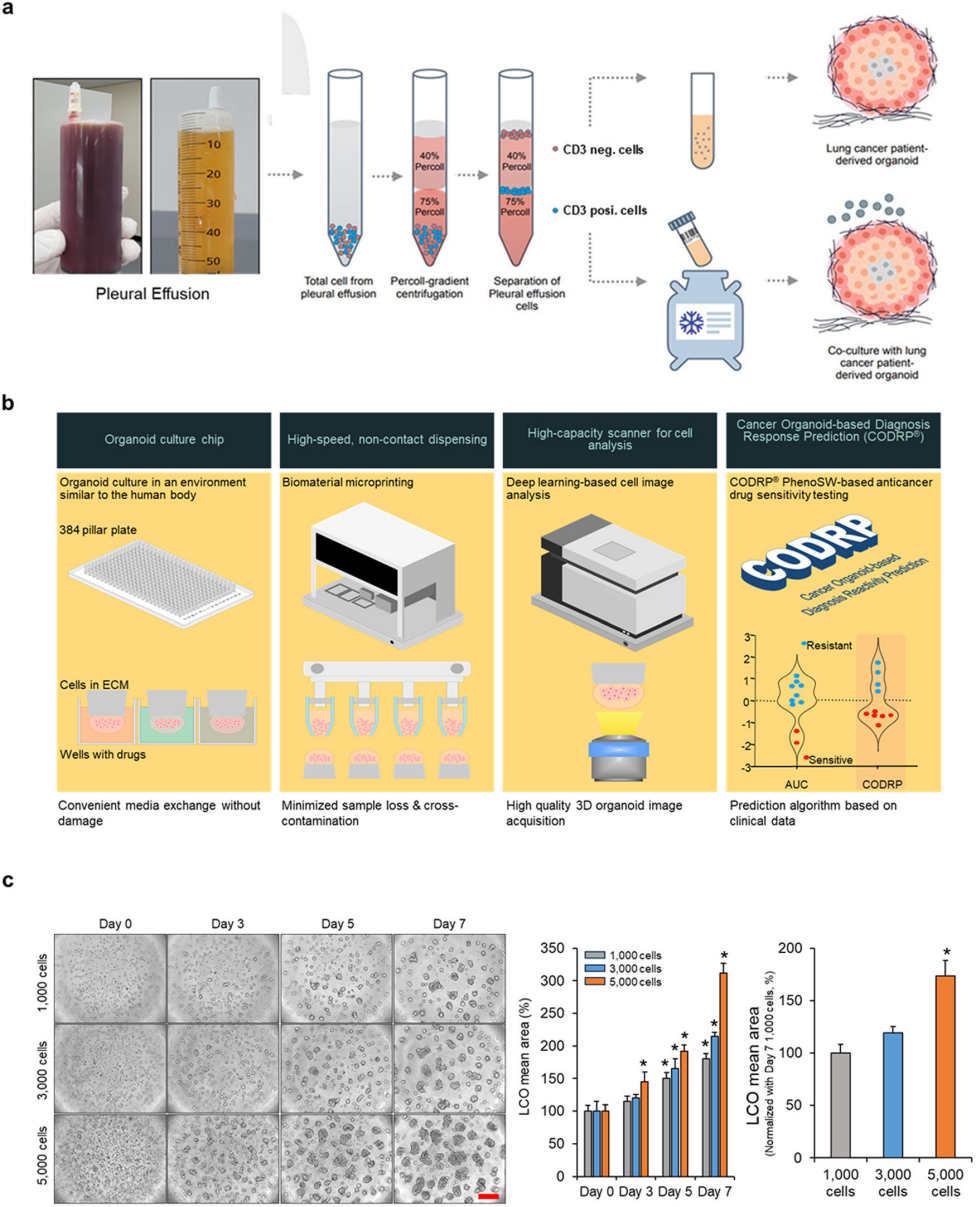
Establishing a precision medicine platform for treating cancer patients. **a** Schematic representation of the method for isolating CD3-negative and CD3-positive cells from PE and their utilization in anticancer drug-sensitive testing. **b** Introduction of CODRP flat form for precision medicine. **c** Cells were loaded using the ASFA® spotter and cultured for indicated days. The Mean area of LCOs was quantified using the ASFA Ez Cell analyzer (MBD). *p < 0.05 compared with Day 0 or 1000 cells. Scale bars: 100 μm.

We used patient-derived cells cultured from the PE of patients with non-small cell lung cancer (NSCLC) to perform anticancer drug sensitivity tests to determine the optimal chemotherapy regimen for patients with lung cancer (Fig. 1a). To confirm the cell distribution in the PE of patients with lung cancer, we performed fluorescence-activated cell sorting (FACS). We observed that PE contained CD45 lineage and EpCAM-positive cells, with CD45 lineage cells (CD19, CD14, CD3, CD8, CD56) accounting for >85% of the total cell population. When these cells were embedded in Matrigel and cultured for 3 days, FACS analysis showed that CD45-positive cells disappeared and EpCAM-positive cells mainely remained (Fig. 1b and Supplementary Fig. 1). These results indicate the short survival rate of CD45 lineage cells. To overcome the short lifespan of these immune cells and assess their sensitivity to immune checkpoint inhibitors (ICIs) using patient-derived immune cells while maintaining the individual’s immune system, cells obtained from the PE were separated using Percoll-gradient centrifugation^11^. FACS analysis was conducted to assess CD3 expression levels in cells from the top and middle layers of the Percoll solution. The results revealed that CD3-positive cells were present in the middle layer but absent in the top layer (Supplementary Figs. 2 and 3). To separate CD3-positive and -negative cells through Percoll-gradient centrifugation, CD3-negative cells were cultured and subjected to anticancer drug sensitivity testing. CD3-positive cells were cryopreserved for ICI sensitivity testing, which was performed by coculturing them with CD3-negative cells (Fig. 2a and Supplementary Fig. 4).

### CODRP generation and establishment

Over the past few years, we created a comprehensive platform for building a precision medicine pipeline that can effectively enhance patient care. This platform consists of several essential components that have been carefully developed to meet individualized medical needs. First, we developed a specialized chip that enabled the cultivation of small quantities of patient-derived cells. These cells were carefully embedded within hydrogels, such as Matrigel^8^. Second, we designed a disposable nozzle-type cell dispenser, facilitating the rapid and precise distribution of cells^8,9^. Additionally, we engineered a scanner with fluorescence-based imaging capabilities to measure drug responses accurately^10^. Finally, we devised advanced algorithms for predicting individualized drug responses to establish a reliable platform for accurately anticipating patient-specific drug reactions (Fig. 2b).

To demonstrate the platform technology, we used an ASFA® spotter to embed CD3-negative cells isolated from PE in Matrigel and then seeded them on Cellvitro® 384 pillars (MBD Co., Ltd.). We observed cell- and culture-time-dependent formation of LCOs, confirming the successful generation of LCO from dispensed cells (Fig. 2c and Supplementary Fig. 5).

### EGFR-TKI drug screening of LCOs from malignant pleural effusion

**Fig. 3:**
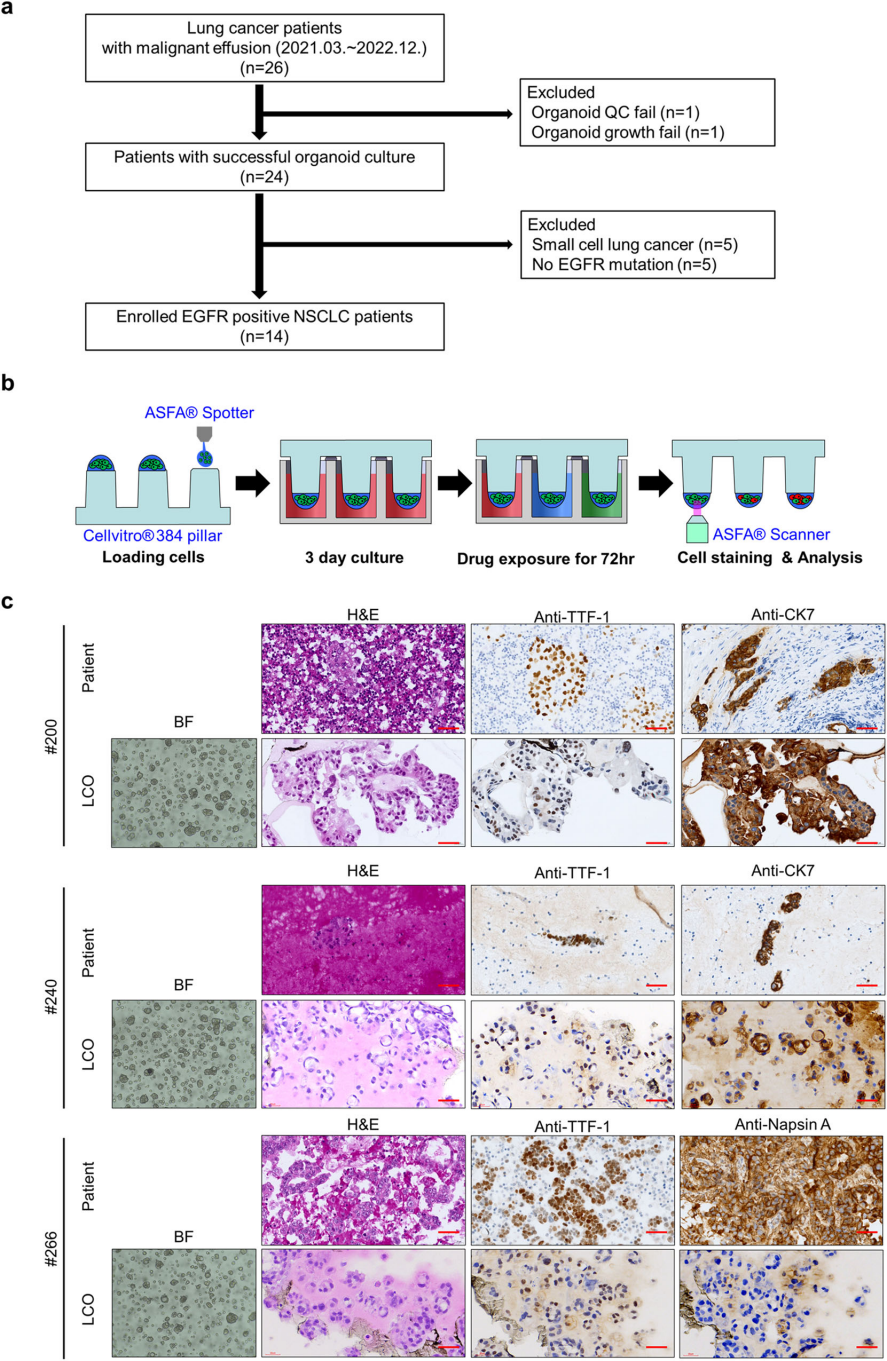
Establishment of LCOs and drug testing platform by CODRP. **a** Overview of the patient selection process. **b** Schematic workflow of CODRP platform using LCOs. **c** Representative brightfield images of LCOs after 7–10 days of culturing in LCOs growth media. H&E and IHC for the indicated antibodies of NSCLC patients PE samples or tissue specimens, as well as on the derived LCOs. Scale bars: 100 μm.

A diagnostic assay that can provide results within 7 days of diagnosis would be useful in clinical decision making, as treatment decisions are often made within this timeframe. This study included patients with lung cancer with malignant PE who visited St. Mary’s Hospital in Seoul between March 2021 and December 2022 and underwent PE drainage. During this period, pleural effusion samples were collected from all 26 patients. Patients with small-cell lung cancer, no EGFR mutations, organoid growth, or quality control (QC) failures were excluded from the analysis. The study ultimately included 14 patients with confirmed EGFR mutations in NSCLC, all of whom received tyrosine kinase inhibitor (TKI) therapy (Table 1 and Fig. 3a).

**Table 1:** Clinical characteristics of the patients

	Sex	Age	Smoking	Diagnosis	Stage at diagnosis	Stage at sampling	Mutation
200	Male	68	Current smoker	Adenocarcinoma	IVB (T4N3M1c)	IVB (T4N3M1c)	EGFR exon19 deletion
224	Male	64	Ex-smoker	Adenocarcinoma	IVB (T3N1M1c)	IVB (T4N3M1c)	EGFR exon19 deletion, T790M
240	Female	56	Never smoker	Adenocarcinoma	IVA (T4N3M1a)	IVB (T4N3M1c)	EGFR exon19 deletion, T790M, ALK translocation
246	Female	65	Ex-smoker	Adenocarcinoma	IVA (T4N1M1a)	IVA (T4N1M1a)	EGFR exon19 deletion, T790M
263	Female	79	Never smoker	Adenocarcinoma	IVB (T3N3M1c)	IVB (T4N3M1c)	EGFR exon19 deletion, T790M
266	Female	61	Never smoker	Adenocarcinoma	IVA (T2aN0M1a)	IVA (T2aN0M1a)	EGFR exon19 deletion, exon20 Insertion
278	Female	49	Never smoker	Adenocarcinoma	IIIB (T4N2M0)	IVB (TXN3M1c)	EGFR exon19 deletion, T790M
282	Female	83	Never smoker	Adenocarcinoma	IIA (T2aN0M0)	IVB (TXN3M1c)	EGFR exon19 insertion, T790M
283	Male	64	Ex-smoker	Adenocarcinoma	IVB (T4N3M1c)	IVB (T4N3M1c)	EGFR exon21 L858R
305	Male	97	Never smoker	Adenocarcinoma	IVB (T4N0M1c)	IVB (T4N0M1c)	EGFR exon21 L858R
316	Male	70	Ex-smoker	Adenocarcinoma	IVB (T1bN0M1c)	IVB (T1bN0M1c)	EGFR exon19 deletion
331	Female	85	Never smoker	Adenocarcinoma	IVA (T2bN2M1a)	IVA (T2bN2M1a)	EGFR exon21 L858R
334	Male	62	Ex-smoker	Adenocarcinoma	IVA (T1bN0M1a)	IVA (T1bN0M1a)	EGFR exon19 deletion
340	Male	65	Ex-smoker	Adenocarcinoma	IVB (T2bN3M1c)	IVB (T4N3M1c)	EGFR exon19 deletion

Of the 14 patients enrolled, seven (50%) were male, and seven (50%) were never-smokers (Table 1). All patients were confirmed to have an adenocarcinoma component in their tissue samples and no cases of squamous cell carcinoma were observed. Twelve (86%) patients were diagnosed at stage 4 and immediately started on EGFR TKI treatment, whereas two (14%) patients were started on TKI therapy after recurrence following definitive treatment (surgery or CCRT). In Korea, third-generation EGFR-TKIs have not yet been approved as first-line therapy for patients with NSCLC with EGFR mutations. Therefore, all patients were administered either first- or second-generation TKIs as the first-line treatment. On confirmation of disease progression, patients with detected T790M mutations in re-biopsy samples transitioned to third-generation EGFR-TKIs (either Osimertinib or Lazertinib), whereas those without T790M mutations transitioned to standard chemotherapy. In this study, 11 (79%) patients were found to have an EGFR exon 19 deletion and three (21%) had an EGFR exon 21 L858R mutation. All patients were treated with second-generation EGFR TKIs; specifically, 13 received afatinib and one received dacomitinib. After disease progression and identification of an exon 20 T790M mutation were confirmed, six patients were administered third-generation EGFR-TKIs. Of these, three patients were administered osimertinib and the remaining three received Lazertinib (Table 2).

**Table 2:** Treatments and clinical responses of NSCLC patients

	Chemotherapy regimen and response according to RECIST 1.1								Organoid drug screening	
	1st line		2nd line		$\ge$ 3rd line				Afatinib	Osimertinib
200	-Sampling time-		Pemetrexed cisplatin	PD					Sensitive	Sensitive
	Afatinib	PR								
224	Afatinib	PR	Osimertinib	PD	-Sampling time-				Resistance	Resistance
					Gemcitabine-Carboplatin	PD				
240	Afatinib	PR	Alectinib	PD	Osimertinib	PR	-Sampling time-		Resistance	Resistance
							Etoposide-Cisplatin	PR		
							Atezolizumab	PD		
246	Afatinib	PR	-Sampling time-						Resistance	Sensitive
			Osimertinib	PR						
263	Afatinib	PR	-Sampling time-						Resistance	Sensitive
			Lazertinib	NE						
266	-Sampling time-								Sensitive	Sensitive
	Afatinib	PR								
278	Afatinib	PR	-Sampling time-						Resistance	Sensitive
			Lazertinib	PR						
282	Gefitinib	PR	Afatinib	PD	-Sampling time-				Resistance	Resistance
					Lazertinib	PD				
283	Dacomitinib	PR	-Sampling time-						Resistance	Resistance
			Pemetrexed-Carboplatin	PD						
305	-Sampling time-								Sensitive	Sensitive
	Afatinib	PR								
316	-Sampling time-								Sensitive	Sensitive
	Afatinib	SD^a^	Tumor decrease: 20%							
331	-Sampling time-								Resistance	Resistance
	Afatinib	SD^a^	Tumor decrease: 10%							
334	-Sampling time-								Sensitive	Sensitive
	Afatinib	PR								
340	Afatinib	PR	-Sampling time^b^-						Sensitive	Sensitive

*PR* partial response, *PD* progressive disease, *SD* stable disease, *NE* not evaluable.

^a^According to RECIST 1.1, the case is classified as SD. However, a follow-up chest CT showed a reduced lung mass size, and the patient has maintained this without disease progression. This case is considered to be sensitive to afatinib.

^b^The patient initially showed a partial response (PR) to afatinib treatment but voluntarily discontinued the medication. Following discontinuation, malignant pleural effusion was observed, along with confirmed disease progression. Subsequently, the patient died due to disease progression.

Using the Perocoll-gradient centrifugation method, we isolated cells from the PE of 14 patients. Subsequently, we employed the CODRP platform using 14 different types of generated LCOs, as described in Fig. 2b, to conduct a study aimed at obtaining drug sensitivity results within 1 week (Fig. 3b). To validate the organoids and compare their morphology and pathology with those of the original patient samples, we performed Hematoxylin-eosin (H&E) staining and immunohistochemistry (IHC). Classic adenocarcinoma (ADC) markers were used, including cytokeratin 7 (CK7), thyroid transcription factor 1 (TTF-1), and napsin A. The results of H&E staining and IHC indicated that the LCOs retained the histopathological characteristics of the original tumor tissue or malignant pleural effusion. For instance, ADC-derived LCOs were observed in clusters exhibiting subtle cytological features such as prominent nucleoli and cuboidal nuclear morphology. These LCOs formed acinar structures that closely resembled those of primary patient samples (Fig. 3c).

### Comparison of AUC and CODRP index for drug sensitivity analysis

Selecting an appropriate anticancer drug for patients with NSCLC with frequent EGFR mutations targeted by second- and third-generation agents presents considerable challenges. Although third-generation agents have been reported to exhibit higher or comparable treatment response rates compared to first- and second-generation agents, second-generation agents have also shown substantial efficacy^12,13^. In this study, we aimed to identify patients who are sensitive to EGFR anticancer agents using LCOs. To this end, we compared the drug sensitivity results derived from the CODRP index, which considers both the existing drug response-based AUC and patient-specific cell growth rates from LCOs, with the conventional AUC index-based clinical outcomes. The goal was to assess whether there were any differences in the matching rates between these two approaches for selecting patients who were sensitive to EGFR anticancer agents.

**Fig. 4:**
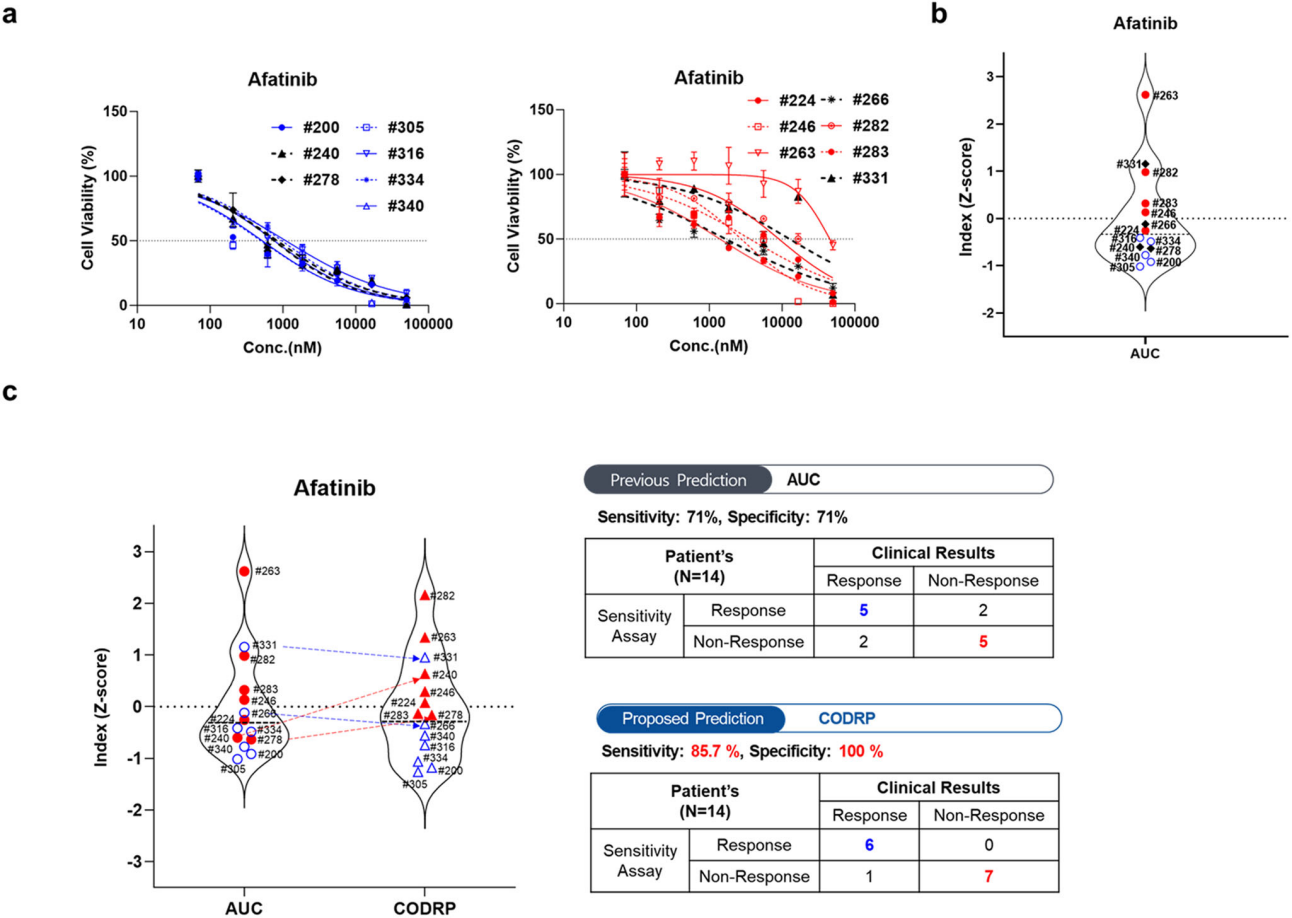
LCOs-based high-throughput screening (HTS) and CODRP index analysis. **a** A total of 5000 cells of LCOs, mixed with Matrigel at an 80% concentration, were spotted on the 384-micropillar surface. LCOs were exposed to Afatinib for 72 h. Subsequently, the mean area was determined by fluorescence intensity, and intracellular ATP levels were assessed. **b** The Z-score values for drug response were calculated based on different means and standard deviations. **c** Comparative analysis of drug response based on the AUC and CODRP indices for Afatinib; the CODRP index takes into account the LCOs growth rate, and it is calculated as a Z-score value based on the different mean and standard deviation (SD) values. Cut-off values for classifying drug responses into sensitive and resistant groups were identified for Afatinib. Cut-off: −0.17.

Drug sensitivity testing for afatinib, a second-generation agent, using LCOs was performed in patients with lung cancer (Fig. 4a and Supplementary Fig. 6). We quantitatively analyzed the sensitivity of afatinib using the AUC values of the dose-response curves (DRCs). The AUC represents an absolute measure of drug efficacy, and the drug index indicates the relative efficacy within the tested patient population. We calculated the AUC index for afatinib in individual patients using the Z-scoring AUC values (Supplementary Table 1). When comparing this index with the clinical outcomes, we observed opposing results in four samples (#240, #266, #278, and #331), as indicated by the black dots (Fig. 4b). Upon incorporating the sample-specific cell growth rate index into the CODRP index, we observed consistency with clinical outcomes in all samples, except for #331 (Fig. 4c). Moreover, upon comparing the CODRP index for the third-generation anticancer agent osimertinib with clinical outcomes, we observed that it exhibited better alignment than the AUC index (Fig. 6 and Supplementary Fig. 7).

### CODRP rapidly and accurately assesses patient drug response in a prospective clinical study

Patient responses to anticancer drugs were evaluated in accordance with the response evaluation criteria in solid tumors (RECIST 1.1) guidelines^14,15^. Imaging studies, including chest computed tomography (CT) scans, were conducted at 3-month intervals to assess treatment response. If a patient exhibited a considerable volume of pleural effusion requiring drainage, either at the initial diagnosis or during the follow-up period after TKI therapy, sampling was performed using a pigtail catheter. Cytological analyses were also conducted on the effusion samples to confirm malignant effusion.

After sampling the malignant pleural effusion, organoid culture was initiated. Drug sensitivity analyses were conducted on cultured LCOs, utilizing both the AUC and the CODRP index for evaluation. The CODRP index incorporates drug sensitivity assessment by considering both the growth rate of LCOs and the AUC. Patients were prospectively monitored, and their responses to the administered TKIs were assessed using RECIST 1.1 criteria and then compared with the LCO drug sensitivity results. Even if the tumor diameter did not decrease by 30% or more, it was classified as stable disease (SD) under RECIST criteria. A clinical response to TKI was determined if a reduction in tumor size was observed. Conversely, even if the tumor size did not increase by 20% or more, increase in tumor size was interpreted as indicative of resistance to TKI therapy.

**Fig. 5:**
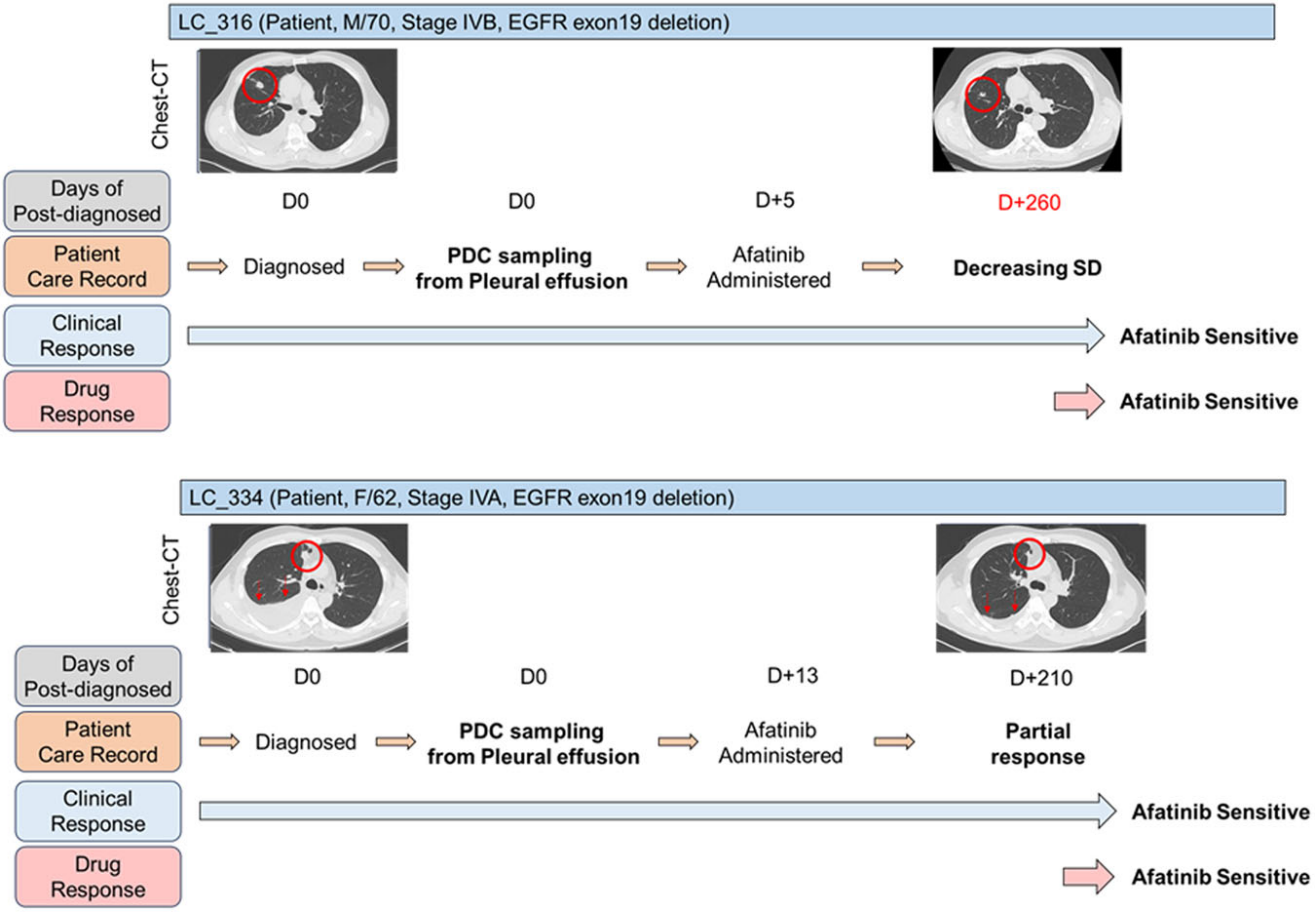
Clinical relevance of LCOs-based HTS analysis and CODRP index analysis. Patient #316 was diagnosed as a stage IVB lung cancer patient with an EGFR exon19 deletion. After treatment with Afatinib, a noticeable trend of size reduction was observed. Consequently, the drug sensitivity test using #316 LCOs derived from PE showed a sensitive response to Afatinib. Patient #334, diagnosed with stage IVA lung cancer and bearing an EGFR exon19 deletion, exhibited a significant reduction in lesions after Afatinib treatment. The drug sensitivity test confirmed a strong positive response to Afatinib.

Case #316: In the present case, pleural effusion was observed at diagnosis and a reduction in tumor size (from 1.5 cm to 1.2 cm, a 10% decrease in tumor size) was observed after administration of afatinib targeting an EGFR exon 19 deletion. Although the patient did not achieve PR according to the RECIST criteria and was categorized as SD, a reduction in tumor size was noted. When assessed using the World Health Organization (WHO) criteria, the tumor response is evaluated using a bidimensional measurement, typically by assessing the product of the longest diameter and its perpendicular diameter for each tumor; these findings correspond to a PR. This difference occurs because the RECIST relies on unidimensional measurements. Although classified as having SD under the RECIST criteria, the patient was examined to be clinically sensitive to afatinib, which is consistent with the results of LCO drug screening (Figs. 4c and 5). Case #334: The patient was initially diagnosed with M1a stage IVA and had an accompanying right-sided effusion at diagnosis. Subsequently, sampling was performed. An EGFR exon 19 deletion was confirmed, and the patient was started on afatinib as first-line TKI therapy. The right upper lobe cancer mass reduced in size from 1.6 cm to 1.0 cm, and the malignant pleural effusion was also improved after afatinib administration. The patient demonstrated partial response (PR) according to the RECIST Criteria in Solid Tumors and maintained PR without evidence of disease progression up to day 210 after afatinib administration. Clinically, it was assessed to be sensitive to afatinib, which was confirmed through drug screening conducted with LCO (Figs. 4c and 5).

**Fig. 6:**
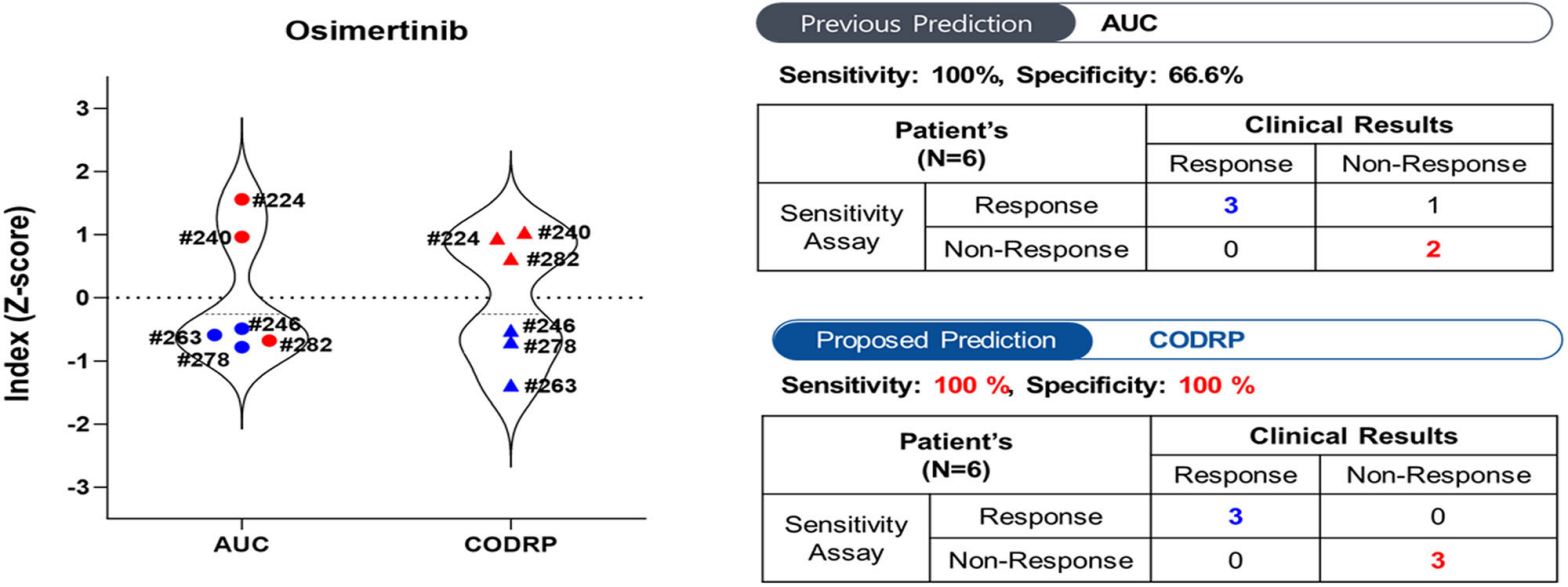
LCOs-based HTS and CODRP index analysis. Comparative analysis of drug response based on the AUC and CODRP indices for Osimetinib; the CODRP index accounts for the LCOs growth rate and is calculated as a Z-score value based on the different mean and standard deviation (SD) values. Cut-off values for classifying drug responses into sensitive and resistant groups were identified for Osimertinib. Cut-off: −0.17.

**Fig. 7:**
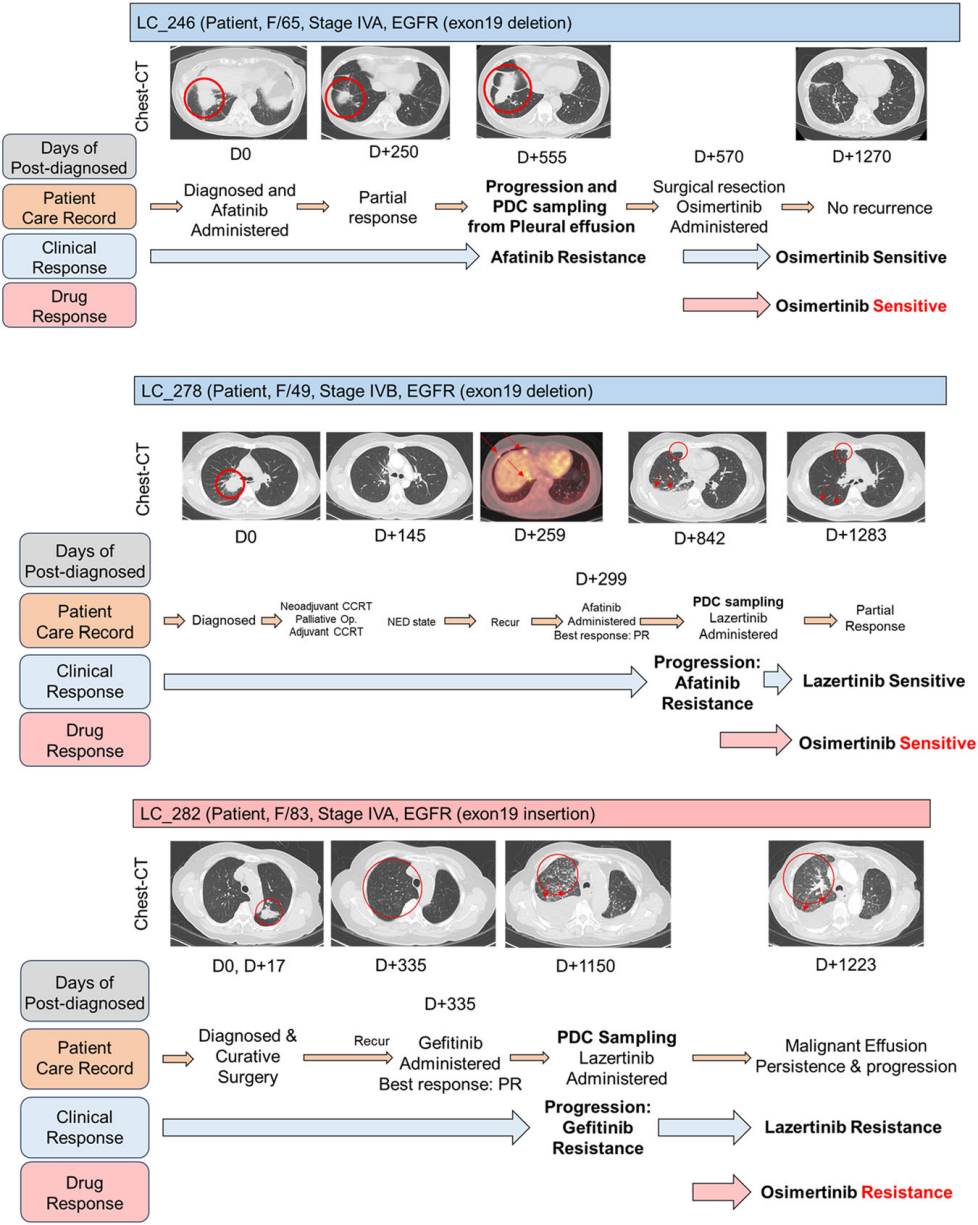
Clinical relevance of LCOs-based HTS analysis and CODRP index analysis. Patients #246 and #278 showed disease progression accompanied by malignant PE during afatinib treatment. Upon disease recurrence, both tissue and malignant PE samples showed the presence of an EGFR exon 20 T790M mutation; consequently, Osimertinib or Lazertinib treatment was initiated. These patients have shown no signs of disease progression while receiving Osimertinib or Lazertinib. Clinically, their response to third-generation TKIs aligns with the findings of the LCO drug screening, suggesting a favorable outcome. Patient #282 experienced disease progression along with the development of malignant PE during gefitinib and afatinib treatment. An EGFR exon 20, T790M mutation was confirmed in PE, leading to the initiation of Lazertinib; however, progressive disease (PD) was observed. Clinically, this case was categorized as resistant to 3rd generation EGFR TKI. Additionally, resistance to the third-generation EGFR TKI, osimertinib, was also noted in LCO drug screening.

Case #246: The patient initially showed a PR to afatinib treatment. However, the patient developed afatinib resistance, leading to disease progression accompanied by malignant pleural effusion on the 555th day. Subsequent restaging and drainage of the malignant pleural effusion were performed, followed by surgical biopsy to secure sufficient tissue for mutation testing of the progressed lung cancer lesion. The patient underwent wedge resection of the right middle and lower lobes, and pleural nodule biopsy. An EGFR exon 20 T790M mutation was identified in both tissue and malignant pleural effusion samples, which led to the initiation of the third-generation TKI osimertinib. The patient is currently undergoing surveillance. Although the target lung cancer lesion was removed via wedge resection, rendering no lesions evaluable for response according to the RECIST, the patient showed no signs of disease progression for over 700 days while being on osimertinib. Clinically, the patient appeared to be sensitive to osimertinib, which aligns with the findings of LCO drug screening. Patient #278 also showed disease progression accompanied by malignant pleural effusion during afatinib treatment, and a mutation in EGFR exon 20, T790M mutation, was confirmed. Consequently, treatment with lazertinib, a third-generation TKI, was initiated. Following the transition to lazertinib, the patient exhibited a PR, allowing for the assessment of sensitivity to third-generation TKIs. This finding is consistent with the results of the LCO drug screening (Figs. 6 and 7). Patients #246 and #278 underwent pleural effusion sampling when disease progression was observed during afatinib treatment. Clinically, this suggested that the patient developed afatinib resistance. The LCO drug screening results for both patients also indicated afatinib resistance (Fig. 4c).

Case #282: The patient was initially diagnosed with stage IIA lung cancer (TNM: T2bN0M0) and underwent curative surgery. On day 335th post-surgery, metastatic tiny nodules were observed in both lungs, indicating disease recurrence. The patient exhibited a near-complete response to gefitinib but experienced relapse on the 1150th day, accompanied by dominant right-sided pleural effusion. Subsequently, the course of treatment was transitioned to afatinib for 1 month; however, disease progression was confirmed. An EGFR exon 20 T790M mutation was confirmed in pleural effusion and re-biopsy samples, leading to the initiation of the third-generation EGFR-TKI lazertinib. During the first response evaluation conducted after 3 months, progressive disease (PD) was observed. Clinically, this case was categorized as resistant to third-generation EGFR-TKI. Additionally, resistance to osimertinib, a third-generation EGFR-TKI, was also noted in the LCO drug screening (Figs. 6 and 7).

Cases #224 and #240: both patients demonstrated disease progression accompanied by pleural effusion after receiving third-generation EGFR-TKI osimertinib. Clinically, they were deemed resistant to the previously administered EGFR TKIs afatinib and osimertinib. This clinical assessment was corroborated by the results of LCO drug screening (Figs. 4c and 6). In case #263, after the initiation of lazertinib treatment, elevated liver enzyme levels and deteriorated patient condition were observed, leading to inconsistent administration of lazertinib. Three months after the initiation of lazertinib, the patient died of acute respiratory distress syndrome (ARDS), which is secondary to COVID-19. Consequently, evaluating the clinical response to third-generation EGFR-TKI is difficult. In patient #340, although a PR to afatinib was observed, the patient voluntarily discontinued the medication. Subsequently, disease progression accompanied by malignant pleural effusion was confirmed. At that time, the malignant pleural effusion and LCO samples were drained. Considering the reinitiation of afatinib, the patient ultimately succumbed to worsening respiratory failure. Assessing the clinical response to afatinib at the time of sampling is challenging.

Based on these findings, our CODRP-based drug sensitivity test is promising for prospectively guiding the selection of suitable anticancer agents.

## Discussion

Approximately 15–30% of patients who are first diagnosed with lung cancer are found to have stage IV with malignant pleural fluid^16,17^. Furthermore, among those already diagnosed at stage IV, approximately 40–50% develop new occurrences of pleural effusion as the disease progresses^18,19^. Histological or cytological confirmation of cancer cells is essential for the pathological diagnosis of lung cancer. When respiratory distress occurs owing to malignant pleural effusion, drainage is performed for diagnostic and therapeutic purposes. This procedure not only alleviates the symptoms of respiratory distress but also enables cancer diagnosis. In patients with lung cancer, a diagnosis can be confirmed by identifying malignant cells in the pleural effusion in more than 40–75% of cases. Specifically, for adenocarcinomas, the diagnostic rate can exceed 90% when assessed cytologically^20^. In such cases, invasive tissue biopsies, such as bronchoscopy, fine-needle aspiration, or even surgery, can be avoided, presenting an advantageous alternative method of diagnosis.

There are distinct advantages in utilizing malignant pleural effusion to cultivate cancer organoids from lung tissue samples. Cancer organoids cultured from lung cancer tissues may be replaced by normal cells^21^. Although several experimental methods have been developed to overcome this issue, they remain challenging. This phenomenon occurs due to the overgrowth of a minority of normal cells within the cancer tissue. However, no normal lung cells were present in the pleural effusion, preventing the emergence of normal organoids. Additionally, although single-cell dissociation is required when culturing organoids from cancer tissues, this step can be skipped when using pleural effusion because the cells are already in a single-cell state. This can reduce the cellular damage during this process.

Recently, there have been reports of studies using droplet chips and microwell array approaches to generate PDOs, including LCOs, from cancer tissues and PE samples. Moreover, some studies have achieved drug sensitivity testing using LCOs within one week^22–24^. However, it is worth noting that the previous studies only compared the drug sensitivity results with clinical outcomes based solely on AUC. In contrast, our current study goes beyond the conventional approach and considers both the AUC and cell growth rate (CODRP index). We observed variations in the growth rates of each LCO, and a negative correlation emerged between the LCO growth rate and AUC value, which represents drug efficacy in the CODRP index drug sensitivity test. Based on these findings, we developed an innovative approach to assess the sensitivity of EGFR-targeted drugs using the CODRP index, which considers both the PDO growth rate and AUC values. Using the CODRP index, we conducted a comprehensive drug sensitivity analysis of EGFR-targeted drugs and successfully differentiated drug sensitivity levels. We observed an enhancement in the matching clinical outcomes for afatinib. Specifically, the sensitivity increased from 71% to 85.7% and the specificity improved to 100% (Fig. 4c). These results emphasize the importance of integrating multiple parameters to enhance personalized treatment decisions for precision medicine.

In precision medicine, TKIs are typically administered at fixed dosages, despite the highly variable outcomes observed in individual patients^25^. According to the findings of this study, AUC values could vary by 2–3 times among LCOs with identical TKI-sensitive driver gene mutations. These contradictory results have sparked interest in incorporating LCOs into therapeutic drug monitoring, although an in-depth understanding of the relationship among AUC values, in vitro pharmacokinetics, and pharmacodynamics remains challenging. In addition, our CODRP platform-based test reflected the acquired resistance to specific TKI therapies in patients. Even in the presence of TKI-sensitive mutations, such as L858R and Exon19 deletion, the drug resistance indicator T790M was absent^26^. This highlights the potential of organoid-based drug sensitivity testing to predict clinical outcomes more accurately than molecular markers.

In South Korea, only first- and second-generation EGFR-targeted agents are approved and covered by health insurance as first-line treatment. Third-generation agents have been approved as non-reimbursed first-line treatments; however, insurance coverage is limited to patients who develop resistance to the T790M mutation during treatment with first- and second-generation EGFR-targeted agents. Although insurance coverage may not be available, improving treatment efficacy using third-line anticancer agents for patients who show no response to first- and second-line therapies could considerably enhance their quality of life. However, predicting which patients will respond to the initial treatments and who will not remains challenging. If a patient with a favorable response to second-line anticancer agents is treated with third-line agents, the development of resistance may hinder opportunities for additional targeted therapies. However, if responsiveness to second-line agents can be determined, second-line treatment can be administered to patients who show a positive response. Subsequently, for those who develop resistance due to the T790M mutation, third-line treatment can be considered, ultimately leading to improved quality of life and better therapeutic outcomes in responsive patients.

**Fig. 8:**
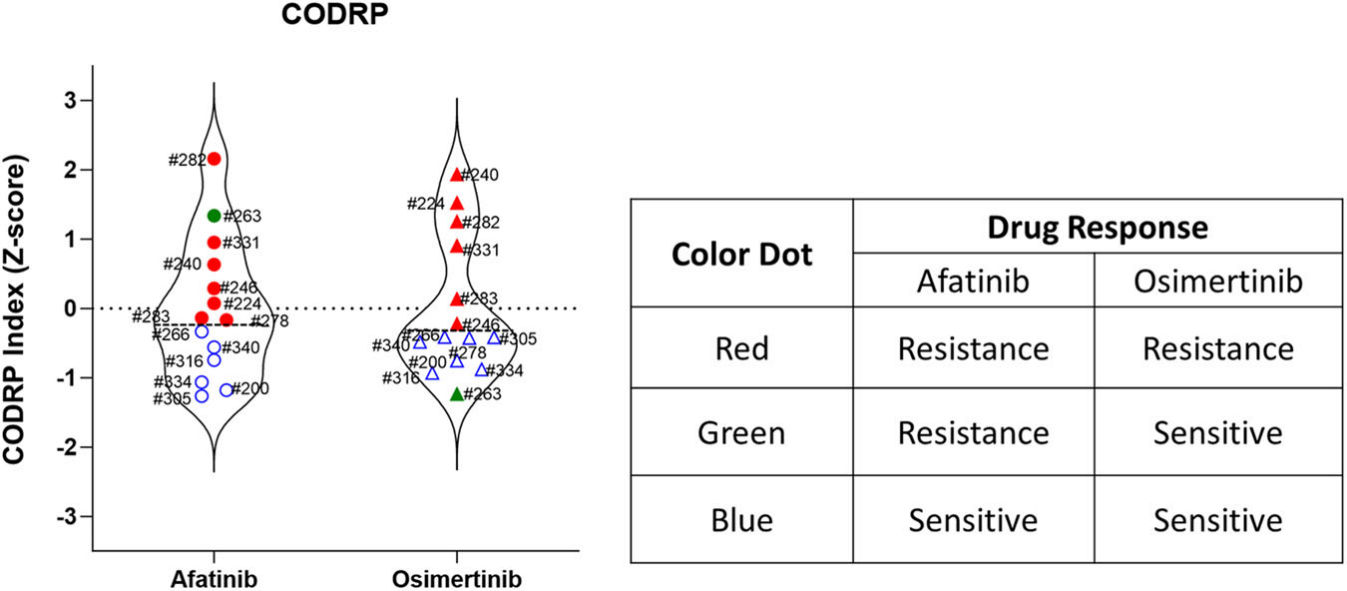
The CODRP platform distinguishes between responders and non-responders to EGFR-TKIs.

Despite the limited number of enrolled patients, our prospective study allowed us to identify individuals (#200, #266, #305, #316, #334, and #340) who exhibited favorable responses to second-generation anticancer agents and to compare their responses with actual clinical outcomes. Furthermore, we predicted that these patients would respond well to third-generation anticancer agents (Fig. 8). Considering these results, using CODRP index for predicting patient treatment responses resulted in a notable improvement compared to the traditional drug response prediction methods limited to AUC or IC50 values.

PD-1 and PD-L1 inhibitors, known as immune checkpoint inhibitors, such as pembrolizumab and atezolizumab, are recommended as standard single-agent or combination therapies for patients without driver gene aberrations. However, the widespread application of PD-1 and PD-L1 inhibitors is hindered by the lack of reliable and dynamic predictive biomarkers^27,28^. Tumor organoids offer a valuable tool for replicating in vivo tumor characteristics; however, their application in immunotherapy research is limited by the absence of an integrated tumor microenvironment. To overcome these limitations, we developed a method involving the coculture of CD3-positive cells, separated using the Percoll-gradient centrifugation technique, with LCOs based on the CODRP platform. Patient #240 initially received afatinib treatment for EGFR exon19 deletion but developed resistance due to the T790M mutation and showed no response to subsequent osimertinib treatment. Therefore, for patient #240, we attempted immunotherapy with atezolizumab, an immune checkpoint inhibitor, owing to low PD-L1 expression. Using the CODRP platform, we evaluated the efficacy of atezolizumab by coculturing CD3-positive and-negative cells isolated from the PE of patient #240. However, we did not observe any significant results, and the clinical outcomes confirmed the lack of efficacy of atezolizumab treatment (Supplementary Fig. 4).

In this study, we developed a novel approach to isolate highly purified cancer and immune cells, specifically CD3-negative cells, from PE samples obtained from patients with lung cancer. The isolated cancer cells were used for drug sensitivity assays using the CODRP index method. The CODRP index demonstrated a remarkable correlation with clinical outcomes, outperforming the conventional approach, which relies solely on the drug response measurement (AUC). These results highlight the potential significance of the CODRP index method, which offers a rapid and accurate assessment of drug sensitivity using pleural effusion-derived cells in patients with lung cancer. This advancement holds promise for predicting drug responses and improving personalized cancer treatment strategies. Additionally, we believe that the utility of the CODRP platform extends beyond EGFR mutations to include other mutations such as ALK, KRAS G12C, and RET fusion, for sensitivity testing of anticancer agents.

## Methods

### Patient sample collection and processing

PE samples were obtained from Seoul St. Mary’s Hospital (Catholic University of Korea), with the written informed consent from patients previously diagnosed with lung adenocarcinoma. This study was conducted following the ethical standards of the Declaration of Helsinki, as well as national and international guidelines. Approval was obtained from the Institutional Review Board of the Catholic University of Korea (IRB NO: KC18TNSI0033).

Each PE sample contained approximately 50–100 mL of fluid, from which we obtained 1 ×10^7^ cells for culture. The samples were transferred from the primary container to a 50-mL centrifuge tube and then centrifuged at 440 × *g* for 10 min at room temperature. The cell pellets were resuspended and washed twice with advanced DMEM/F12 medium. To create a 90% Percoll solution, an appropriate amount of Percoll (density: 1.130 g/mL) was mixed with Hank’s balanced salt solution containing phenol red. Subsequently, 40% and 75% Percoll solutions were prepared by mixing the appropriate amount of 90% Percoll with buffer 1 (consisting of 10% fetal bovine serum +100 μM EDTA in PBS). The MPE samples were strained with a 70-mm cell strainer and centrifuged at 440 × *g* for 10 min at room temperature. The cell pellets were rinsed with the RPMI medium. After centrifugation, each cell pellet was resuspended in a single-cell suspension with 5 mL of 75% Percoll solution and transferred to a 15 mL centrifuge tube. Forty percent Percoll solution was gently added to the 75% Percoll solution and centrifuged at 780 × *g* for 20 min at room temperature without interruption. The cells, separated into top and middle layers owing to their different densities, were transferred to individual tubes and washed with advanced DMEM/F12 medium. CD3-negative cells in the top layer were used for LCO culture and drug sensitivity tests. The pellet containing the cancer cells was collected and cultured in advanced DMEM/F12 medium along with supplements for LCO culture. Additional information regarding the supplements is provided in Supplementary Table 2.

### Calculation of drug response in LCO-based high-throughput screening (AUC Index)

The isolated patients-derived cells (PDCs) were first filtered through a 100 μm strainer and then combined with Matrigel in a mixture containing approximately 5000 cells and 80% Matrigel (80 v/v) per 1.5 μL volume. These PDC-Matrigel mixtures were dispensed onto 384-pillar plates using an ASFA® Spotter, a device that uses a disposable nozzle to deposit 1.5 μL droplets of the PDC-Matrigel mixtures onto the surface of the 384-pillar plate. The PDC-Matrigel mixtures were then sandwiched (or “stamped”) between the 384-pillar plate they were dispensed on and the 384-well plate for LCO culture and drug exposure. The PDC-dispensed 384-pillar plate was combined with a 384-well plate containing the fresh culture medium and pre-cultured for 3 days in a 5% CO_2_-humidified incubator to form LCOs. The EGFR inhibitors (afatinib and osimertinib) were purchased from AdooQ Bioscience (Irvine, CA, USA) and dissolved in a stock solution of 10 mM dimethyl sulfoxide (DMSO). These drugs were dispensed using the non-contact drug fast-dispensing mode of ASFA™ Spotter. A 384-well plate was divided into six regions. Each region comprised a 3 × 7 well array corresponding to the two EGFR-targeted drugs in a three-fold and seven-point serial dilution series from 50 μM (including one DMSO control) and individual drugs were tested under three technical replicate conditions. The 3D-cultured LCOs were incubated at 37 °C in a 5% CO_2_ humidified incubator to be exposed to the EGFR targeted drugs for 3 days. After incubation, the 384-pillar plate in which the LCOs were cultured was combined with a fresh 384-well plate containing a live-cell staining solution for the specific staining of living cells after drug treatment. The staining solution was prepared by adding 4 μM calcein AM (Invitrogen, CA, USA) in DMEM/F12 medium. Cells were incubated with the staining solution for 1 h at 37 °C in a 5% CO_2_ humidified atmosphere. Live cell images with green fluorescence intensities (excitation/emission, 494/517 nm from lasers) were scanned using an optical scanner (ASFA® Scanner, MBD Co., Ltd. South Korea). The scanned images were evaluated using image analysis software (ASFA Ez Cell analyzer, MBD Co., Ltd., South Korea), and the growth rate of LCOs was calculated from days 1–3. After imaging, cell viability was determined using an adenosine triphosphate (ATP) monitoring system based on firefly luciferase (CellTiter-Glo® Cell Viability Assay, Promega, Madison, WI), according to the manufacturer’s protocol. The ATP assay mixture was prepared by adding 20 μL of CellTiter-Glo reagent to 20 μL of the medium per well. The cells were incubated at room temperature for 30 min to stabilize the luminescence signal, which was recorded using a TECAN Spark microplate reader (TECAN, Männedorf, Kanton Zürich, Switzerland). We performed a specific and accurate drug response analysis using DRC according to the concentration gradient (GraphPad Prism 9; GraphPad Software, CA, USA). The graph confirms the response to EGFR-targeted drugs in individual LCOs through quantified AUC indices. The AUC index was converted into a standard score (Z-score). Using the mean and standard deviation of the AUCs of the EGFR-targeted drugs in individual LCOs, the AUC index was calculated as follows:

$${{AUC\; Index}}_{{drug}}={{Z\,score\; of\; AUC}}_{{drug}}=\frac{{{AUC}}_{{drug}}-{mean}}{{SD}}$$

### Calculation of CODRP index (multi-parameter analysis)

The growth rate of LCOs was measured by the mean area of live LCOs that increased during the 3 days of drug treatment and then converted to a Z-score. In addition, the CODRP index was calculated by comprehensively considering the scored growth rate of individual PDO and the AUC value.

The CODRP index was calculated as follows:

$$\begin{array}{ll}{CODRP\; {Index_{drug}}}\\=\sqrt{{\left({Z\,score\; of\; Growth\; Rate}-(-4.5)\right)}^{2}+{\left({Zscore\; of}{{AUC}}_{{drug}}-(-4.5)\right)}^{2}}\end{array}$$

Where, −4.5 indicates the minimum Z-score for which the value is less than 0.003% from a normal distribution and is a reference point for drug sensitivity. The CODRP index is the normalized distance of a case from the reference point. Drug resistance increased with increase in distance from the reference point. Therefore, as suggested in the conceptual diagram of the CODRP algorithm, the CODRP algorithm was proposed to classify sensitivity to EGFR-targeted drugs according to the EGFR mutation-positive and-negative status.

### Experimental protocol of histology and immunostaining

Additional QC samples were prepared to verify whether the PDOs retained oncological characteristics similar to those of the primary tumor. The prepared PDOs for QC were subjected to IHC analysis for pathological analysis, similar to the patient tissue. The isolated PDCs were seeded in a 4-well plate at a concentration of approximately 1 × 10^5^ cells and 80% Matrigel (80 v/v) per 30 μL volume. The plates were inverted and incubated at 4 °C for 10 min. A four-well plate containing the PDCs was transferred and gelled for 60 min at 37 °C in a humidified incubator containing 5% CO_2_. The cells were cultured for approximately 2 weeks until LCOs were formed, and the culture medium was changed every 3 days. The LCOs were fixed, embedded in paraffin, sectioned, and stained. TTF-1 (Cell Signaling Technology, #12373, Beverly, MA, USA, 1:250), napsin A (Cell Signaling Technology, #62434, 1:200), and CK7 (Cell Signaling Technology, #13092, 1:100) were stained using the Bond-III stainer (Leica Microsystems). Hematoxylin and eosin (H&E) and immunohistochemical images were acquired using an Ocus®40 digital microscope scanner (Grundium Oy, Finland).

### Statistical analysis

Statistical analyses were conducted using either Spearman’s correlation coefficient test or paired/unpaired (two-tailed). The software and algorithms for statistical analyses.

### Reporting summary

Further information on research design is available in the Nature Research Reporting Summary linked to this article.

### Supplementary information

Supplementary data

Reporting summary

## Data Availability

All data generated or analyzed during this study are included in this published article.
