# Supplementary material for: Prediction of TKI response in EGFR-mutant lung cancer patients-derived organoids using malignant pleural effusion
Source: NPJ Precis Oncol. 2024 May 21;8:111. doi: 10.1038/s41698-024-00609-7 (PMC11109121; doi:10.1038/s41698-024-00609-7)
Supplement: Supplementary file 1 — Supplementary data [file 41698_2024_609_MOESM1_ESM.pdf]

Supplementary information

Prediction of Tyrosine Kinase Inhibitor Response in EGFR-Mutant Lung Cancer Patients-derived Organoids using Malignant Pleural Effusion

Running title: Organoid-Based Prediction of TKI Response in EGFR-Mutant NSCLC

Sang-Hyun Lee[1,*], Kyuhwan Kim[2,*], Eunyoung Lee[2,*], Kyungmin Lee[1], Kyeong Hwan Ahn[1], Hansom Park[1], Yelim Kim[1], Soeun Shin[1], Sang Youl Jeon[1], Yongki Hwang[2], Dong Hyuck Ahn[2], Yong-Jun Kwon[3], Seok Whan Moon[4], Mi Hyoung Moon[4], Kyung Soo Kim[4], Kwanyong Hyun[4], Tae-Jung Kim[5], Yeoun Eun Sung[5], Joon Young Choi[6], Chan Kwon Park[7], Sung Won Kim[8,9], Chang Dong Yeo[11], Hyun-Jung Sohn[12], You-Seok Hyun[12], Tai-Gyu Kim[12], Bosung Ku[1,†], Jeong Uk Lim[7,†], Seung Joon Kim[2,10,†]

Supplementary Fig. 1

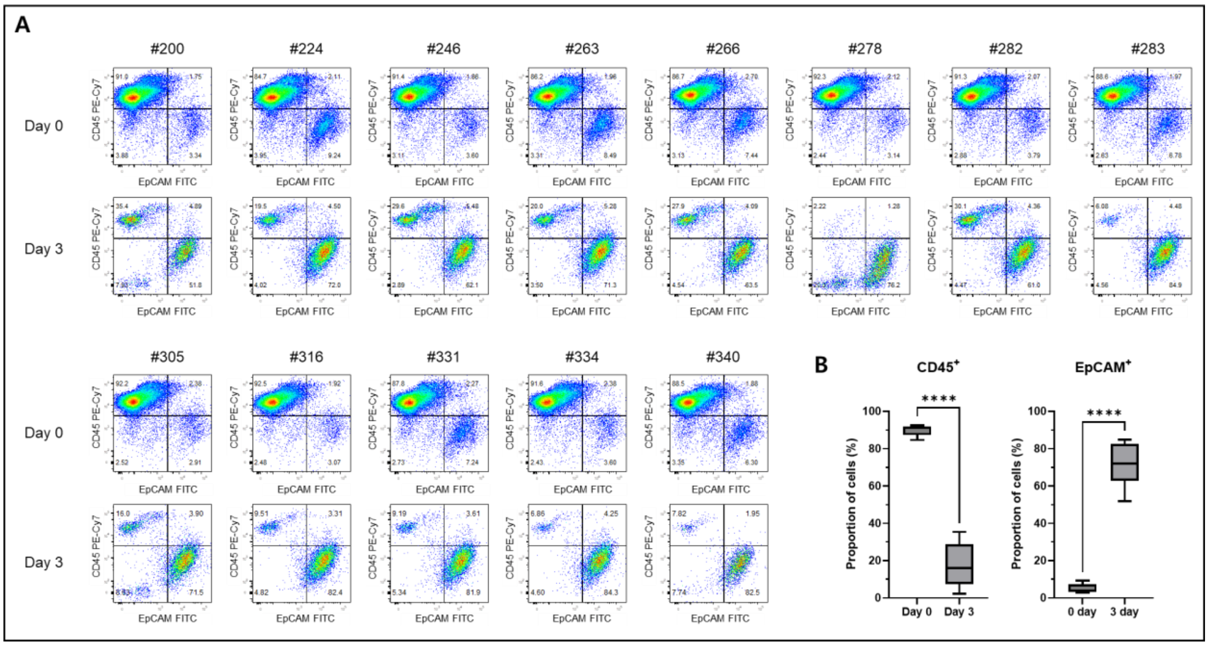

Supplementary Fig. 1 Determination of cellular distribution within pleural effusion using FACS.

Supplementary Fig. 2

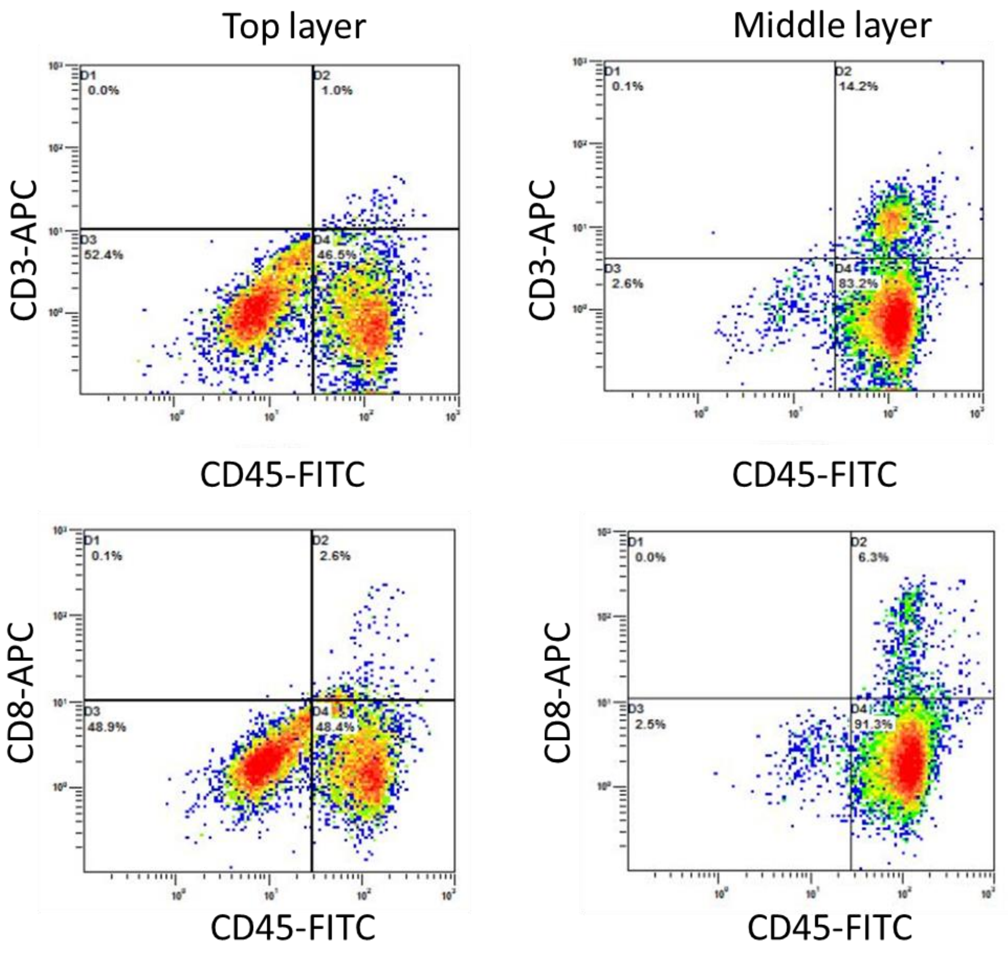

Supplementary Fig. 2 Flow cytometry analysis of CD3-positive and CD3-negative cells. CD3-positive cells were present in the middle layer but were absent in the top layer.

Supplementary Fig. 3

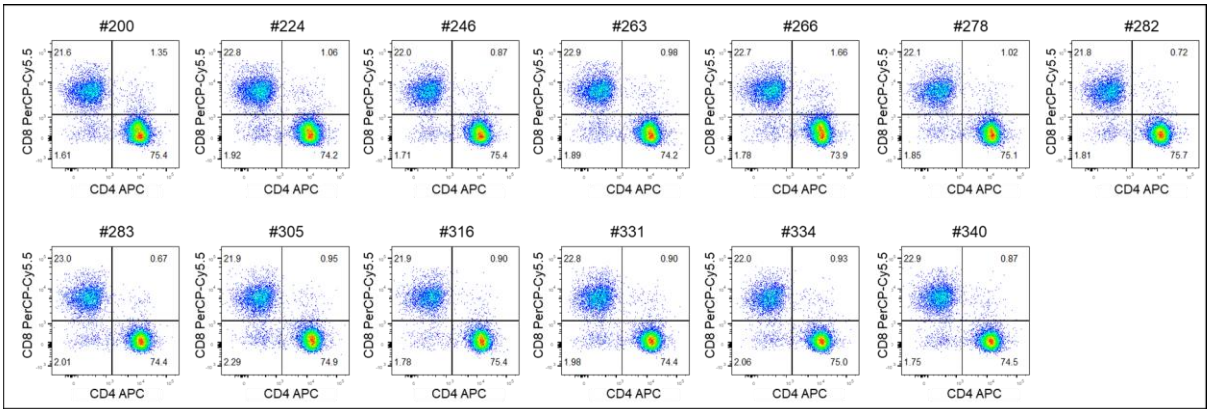

Supplementary Fig. 3 Cell population within the middle layer after Percoll gradient separation.

Supplementary Fig. 4

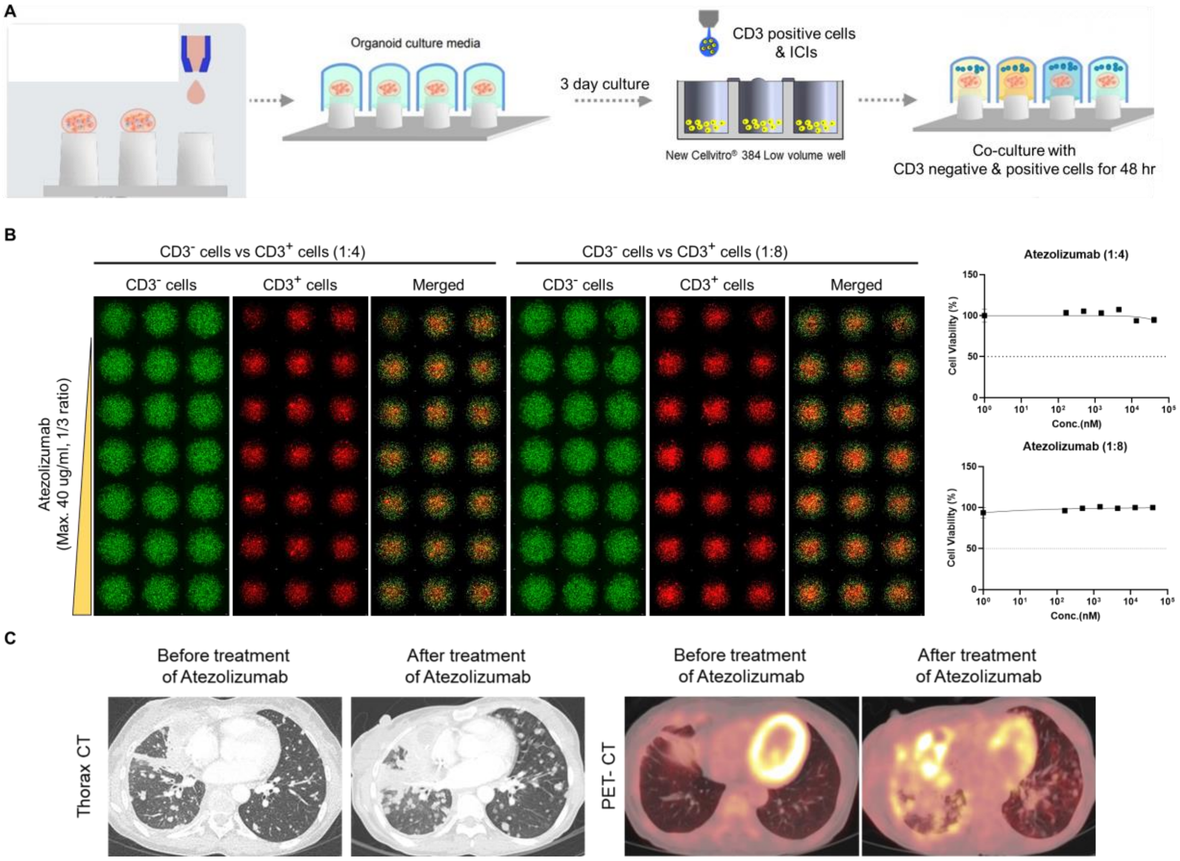

Supplementary Fig. 4 Creation of a platform for testing the sensitivity of immune checkpoint inhibitors. (**A**) Illustration depicting the co-cultivation method of CD3-negative and CD3-positive cells and their utilization in sensitivity testing for immune checkpoint inhibitors. (**B**) CD3-negative cells isolated from patient #240 were loaded into the ASFA® spotter and cultured for 72 hours. After 72 hours, CD3-positive cells were seeded with atezolizumab into a 384-well plate. In a nutshell, they were sandwiched (or "stamped") between the 384-pillar plate on which they were dispensed and the 384-well plate, and then cultured for 48 hours. Following exposure to LCOs with drug and CD3-positive cells, cell viability was measured by fluorescence intensity. (**C**) Chest scan and PET-CT results of patient #240 after Atezolizumab treatment.

Supplementary Fig. 5

A

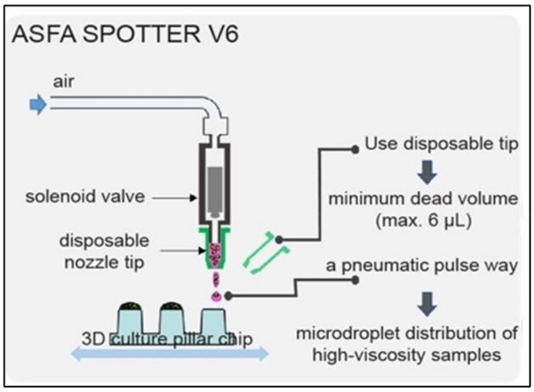

B

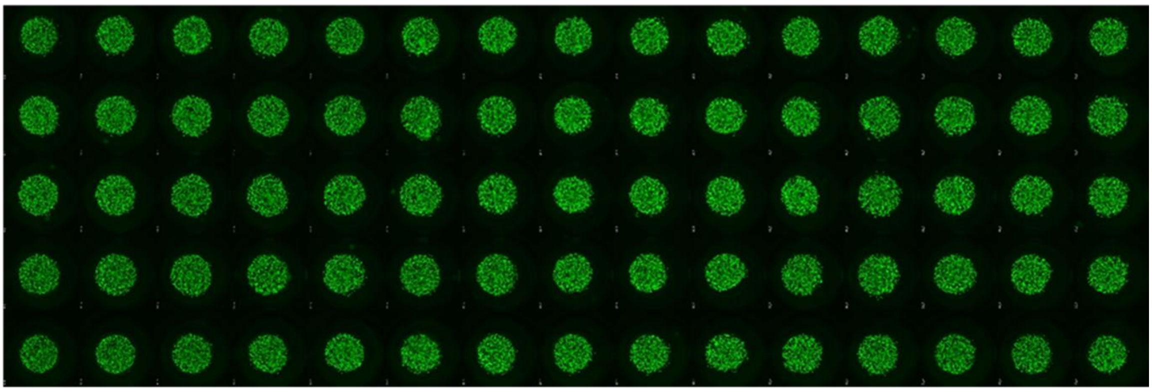

Supplementary Fig.5 (**A**) Schematic structure of ASFA® spotter. (**B**) 5,000 cells of LCOs, mixed with Matrigel at an 80% concentration, were spotted onto the 384-micropillar surface. The images display Calcein AM staining.

Supplementary Fig. 6

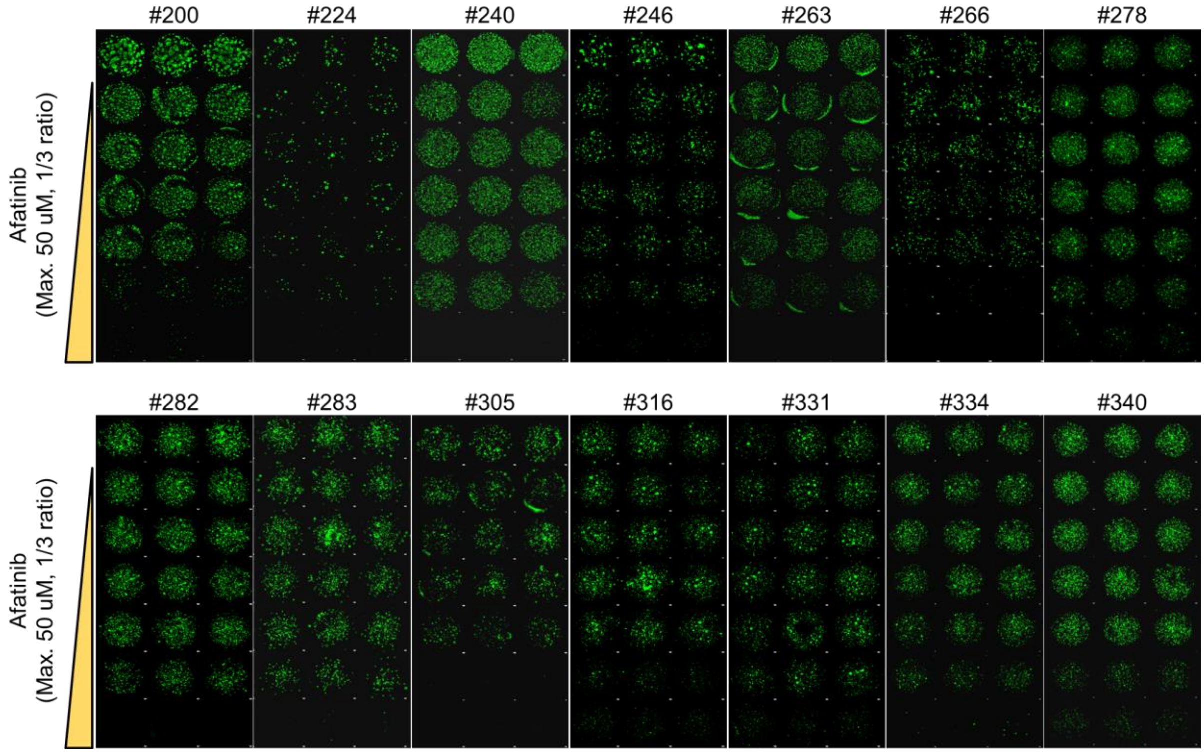

Supplementary Fig.6 5,000 cells of LCOs, mixed with Matrigel at an 80% concentration, were spotted onto the 384-micropillar surface. LCOs were exposed to Afatinib for 72 hr and subsequently, the mean area was determined by fluorescence intensity

Supplementary Fig. 7

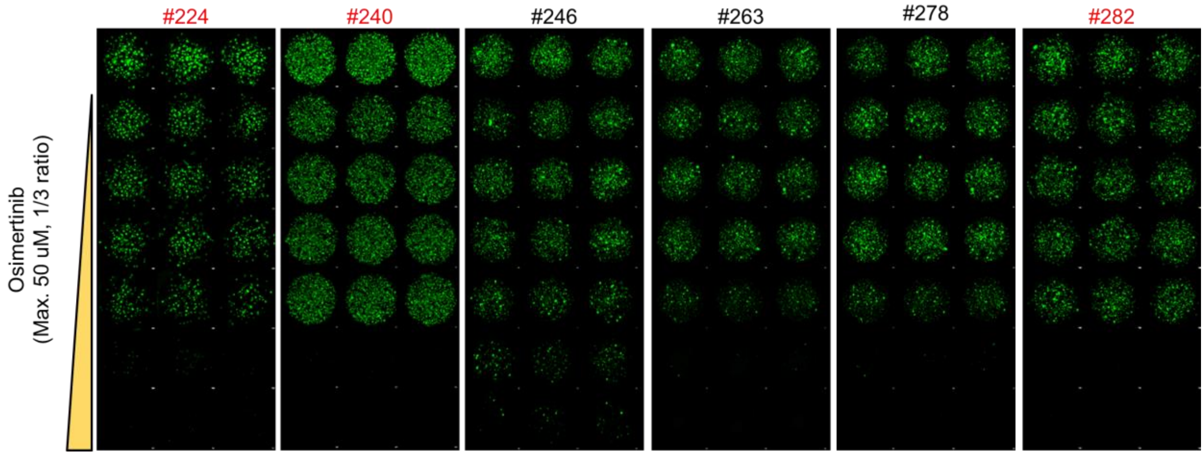

Supplementary Fig.7 5,000 cells of LCOs, mixed with Matrigel at an 80% concentration, were spotted onto the 384-micropillar surface. LCOs were exposed to Ofatinib for 72 hr and subsequently, the mean area was determined by fluorescence intensity

Supplementary Table 1

Supplementary Table 1. AUC and CODRP index of NSCLC patients

Patient	Cell Growth Rate	AUC Index (Z-Score of AUC)		CODRP Index (Z-Score of CODRP)	
		Afatinib	Osimertinib	Afatinib	Osimertinib
#200	118	-0.915	-0.182	-1.179	-0.689
#224	155	-0.257	2.128	0.075	1.678
#240	185	-0.600	1.304	0.632	1.726
#246	154	0.132	-0.711	0.290	-0.211
#263	110	2.619	-0.849	1.338	-1.310
#266	133	-0.118	-0.184	-0.333	-0.378
#278	154	-0.635	-1.120	-0.168	-0.432
#282	212	0.984	-0.975	2.162	1.186
#283	128	0.321	0.761	-0.138	0.176
#305	117	-1.014	0.284	-1.264	-0.377
#316	123	-0.414	-0.776	-0.743	-0.980
#331	153	1.156	1.238	0.952	1.009
#334	110	-0.487	-0.290	-1.062	-0.924
#340	141	-0.773	-0.627	-0.561	-0.475

Supplementary Table 2

Supplementary Table 2. Recipe of the lung cancer organoids culture media

Components	Final Concentration	Source (Vendor)
Advanced DMEM/F12	1X	Gibco
Penicillin streptomycin	100 μg/ml	Gibco
GlutaMAX	1X	Gibco
HEPES	10mM	Gibco
B-27	1X	Gibco
N-acetyl-L-cystein	1.25mM	Sigma
Nicotinamide	5mM	Sigma
R-spondin 3	250ng/ml	Peprotech
Neuregulin 1	5nM	Peprotech
Noggin	100ng/ml	Peprotech
FGF7	5ng/ml	Peprotech
FGF10	20ng/ml	Peprotech
EGF	5ng/ml	Peprotech
A83-01	500nM	Tocris
Y-27632	5μM	AbMole

Supplementary Table 3

Supplementary Table 3. The EGFR mutation of LCOs

Sample		Result	Melt peak(Temperature)						
			E19del/E20insA/EIC				E20insB	T790M	L858R
No.	ID		HEX	ROX	Cy5	Ct	ROX	HEX	ROX
NC		Acceptable	None	None	59	17.97	None	None	None
PC		Acceptable	62	64.5	59.5	14.94	66.5	61.5	57
1	#240	E19del(>1,000)	62.5	None	59	12.52	None	None	None
2	#266	E19del(>1,000)	64	None	60.5	13.11	None	None	None
3	#283	L858R(619)	None	None	60.5	12.56	None	None	56.5
4	#305	L858R(96)	None	None	60.5	14.84	None	None	56.5
5	#331	L858R(92)	None	None	60	14.59	None	None	56.5
