# Supplementary material for: Prediction of TKI response in EGFR-mutant lung cancer patients-derived organoids using malignant pleural effusion
Source: NPJ Precis Oncol. 2024 May 21;8:111. doi: 10.1038/s41698-024-00609-7 (PMC11109121; doi:10.1038/s41698-024-00609-7)
Supplement: Supplementary file 2 — Reporting summary [file 41698_2024_609_MOESM2_ESM.pdf]

nature portfolio

Corresponding author(s): Professor Seung Joon Kim

Last updated by author(s): Feb 20, 2024

Reporting Summary

Nature Portfolio wishes to improve the reproducibility of the work that we publish. This form provides structure for consistency and transparency in reporting. For further information on Nature Portfolio policies, see our Editorial Policies and the Editorial Policy Checklist.

Statistics

For all statistical analyses, confirm that the following items are present in the figure legend, table legend, main text, or Methods section.

n/a	Confirmed	
☐	☒	The exact sample size (*n*) for each experimental group/condition, given as a discrete number and unit of measurement
☐	☒	A statement on whether measurements were taken from distinct samples or whether the same sample was measured repeatedly
☐	☒	The statistical test(s) used AND whether they are one- or two-sided<br>*Only common tests should be described solely by name; describe more complex techniques in the Methods section.*
☐	☒	A description of all covariates tested
☐	☒	A description of any assumptions or corrections, such as tests of normality and adjustment for multiple comparisons
☐	☒	A full description of the statistical parameters including central tendency (e.g. means) or other basic estimates (e.g. regression coefficient) AND variation (e.g. standard deviation) or associated estimates of uncertainty (e.g. confidence intervals)
☐	☒	For null hypothesis testing, the test statistic (e.g. *F*, *t*, *r*) with confidence intervals, effect sizes, degrees of freedom and *P* value noted<br>*Give P values as exact values whenever suitable.*
☐	☒	For Bayesian analysis, information on the choice of priors and Markov chain Monte Carlo settings
☐	☒	For hierarchical and complex designs, identification of the appropriate level for tests and full reporting of outcomes
☐	☒	Estimates of effect sizes (e.g. Cohen's *d*, Pearson's *r*), indicating how they were calculated

Our web collection on statistics for biologists contains articles on many of the points above.

Software and code

Policy information about availability of computer code

Data collection	Excel office 2019, GraphPad Prism Version 10.1.2, Adobe Photoshop CS
Data analysis	Excel office 2019, GraphPad Prism Version 10.1.2

For manuscripts utilizing custom algorithms or software that are central to the research but not yet described in published literature, software must be made available to editors and reviewers. We strongly encourage code deposition in a community repository (e.g. GitHub). See the Nature Portfolio guidelines for submitting code & software for further information.

Data

Policy information about availability of data

All manuscripts must include a data availability statement. This statement should provide the following information, where applicable:

- Accession codes, unique identifiers, or web links for publicly available datasets
- A description of any restrictions on data availability
- For clinical datasets or third party data, please ensure that the statement adheres to our policy

n/a

Research involving human participants, their data, or biological material

Policy information about studies with human participants or human data. See also policy information about sex, gender (identity/presentation), and sexual orientation and race, ethnicity and racism.

Reporting on sex and gender	n/a
Reporting on race, ethnicity, or other socially relevant groupings	n/a
Population characteristics	n/a
Recruitment	This study included patients with lung cancer with malignant PE who visited St. Mary's Hospital in Seoul between March 2021 and December 2022 and underwent PE drainage.
Ethics oversight	This study was approved by the Institutional Review Board of the Catholic University of Korea (IRB: KC18TNSI0033).

Note that full information on the approval of the study protocol must also be provided in the manuscript.

Field-specific reporting

Please select the one below that is the best fit for your research. If you are not sure, read the appropriate sections before making your selection.

☒ Life sciences ☐ Behavioural & social sciences ☐ Ecological, evolutionary & environmental sciences

For a reference copy of the document with all sections, see nature.com/documents/nr-reporting-summary-flat.pdf

Life sciences study design

All studies must disclose on these points even when the disclosure is negative.

Sample size	Use of 100ml pleural effusion in patients with malignant pleural effusion accompanying non-small cell lung cancer.
Data exclusions	During this period, pleural effusion samples were collected from all 26 patients. Patients with small-cell lung cancer, no EGFR mutations, organoid growth, or quality control (QC) failures were excluded from the analysis.
Replication	n/a
Randomization	Classified according to response to EGFR-targeted anticancer agents.
Blinding	It was conducted through a blinding test without knowing individual patient responses to EGFR-targeted anticancer agents in actual clinical results.

Reporting for specific materials, systems and methods

We require information from authors about some types of materials, experimental systems and methods used in many studies. Here, indicate whether each material, system or method listed is relevant to your study. If you are not sure if a list item applies to your research, read the appropriate section before selecting a response.

Materials & experimental systems

n/a	Involved in the study	
☐	☒	Antibodies
☒	☐	Eukaryotic cell lines
☒	☐	Palaeontology and archaeology
☒	☐	Animals and other organisms
☒	☐	Clinical data
☒	☐	Dual use research of concern
☒	☐	Plants

Methods

n/a	Involved in the study	
☒	☐	ChIP-seq
☐	☒	Flow cytometry
☒	☐	MRI-based neuroimaging

Antibodies

Antibodies used	BD FITC Mouse Anti-Human EpCAM (#347197), BD PE-Cy7 Mouse Anti-Human CD45 (#557748), BD PerCP-Cy5.5 Mouse Anti-Human CD8 (#560662), BD APC Mouse Anti-Human CD4 (#561841)

	BD PE Mouse Anti-Human CD19 (#555413), BD FITC Mouse Anti-Human CD14 (#555397) BD BV421 Mouse Anti-Human CD3 (#563797), Anti-TTF1 antibody (Abcam, ab204411), Anti-Cytokeratin 7 antibody (Abcam, ab154334), Anti-Napsin A antibody (Abcam, ab73021)
Validation	n/a

Plants

Seed stocks	n/a
Novel plant genotypes	n/a
Authentication	n/a

Flow Cytometry

Plots

Confirm that:

☒ The axis labels state the marker and fluorochrome used (e.g. CD4-FITC).

☒ The axis scales are clearly visible. Include numbers along axes only for bottom left plot of group (a 'group' is an analysis of identical markers).

☒ All plots are contour plots with outliers or pseudocolor plots.

☒ A numerical value for number of cells or percentage (with statistics) is provided.

Methodology

Sample preparation	After binding cells obtained from pleural effusion of lung cancer patients to antibodies, FACS analysis was performed.
Instrument	BD FACSymphony A3
Software	BD FACSDiva
Cell population abundance	CD45 lineage cells and EpCAM positive cells
Gating strategy	1. Initial gating: Initial gates were set based on cell size and complexity using FSC (Forward Scatter) and SSC (Side Scatter).<br>2. Single-cell gating: Dual-trigger gating was performed to select only single cells within the initial gate.<br>3. Background noise removal: Gates were set using appropriate Fluorescence Minus One (FMO) controls to remove background noise.<br>4. Determination of positive and negative cell populations: Dual-color or single-color analysis was performed to determine positive and negative cell populations for specific antibodies, and appropriate gates were set.<br>5. Gate validation: Gates were validated and adjusted using FMO controls to ensure proper gating throughout the analysis.

☒ Tick this box to confirm that a figure exemplifying the gating strategy is provided in the Supplementary Information.
